# Small Session Size and Big Vial Size: Operational Research Assessing Open Vial Vaccine Wastage at the Service Delivery Points in the Mandalay Region of Myanmar During 2018

**DOI:** 10.3390/tropicalmed5020060

**Published:** 2020-04-15

**Authors:** Aung Naing Oo, Pruthu Thekkur, Aye Mya Cha Thar, Kyaw Ko Ko Htet, Htar Htar Lin

**Affiliations:** 1 Expanded Programme on Immunization, Ministry of Health and Sports, Nay Pyi Taw 15011, Myanmar; dr.achan84@gmail.com (A.M.C.T.); dr.htarhtarlin@gmail.com (H.H.L.); 2 Centre for Operational Research, International Union Against Tuberculosis and Lung Disease (The Union), 75006 Paris, France; pruthu.tk@theunion.org; 3 The Union South-East Asia Office, New Delhi 110016, India; 4 Department of Medical Research (Pyin Oo Lwin Branch), Ministry of Health and Sports, Pyin Oo Lwin 05081, Myanmar; kyawkkhtet@gmail.com

**Keywords:** vaccine wastage, vaccine utilization, EPI, service delivery point, Myanmar

## Abstract

The World Health Organization (WHO) recommends immunization programmes to monitor vaccine wastage at storage and service delivery points. As there were no vaccine wastage assessments in Myanmar, we aimed to assess the vaccine wastage rates in the Mandalay region. We conducted a cross-sectional descriptive study with the inclusion of all immunization sessions conducted through the twenty randomly selected subcentres in the year 2018. The wastage rates were calculated by aggregating vaccine utilization data from selected subcentres. The vaccine wastage rates for Bacillus Calmette–Guérin (BCG) (54.9%), inactivated polio vaccine (28.3%), and measles-rubella (27.4%) were higher than the WHO indicative rates. The high vaccine wastage rates were seen in lyophilized vaccines (36.9%), vaccines requiring only a single dose per child for complete immunization (39.1%), and those with a large vial size of 20 doses (38.8%). The median session size for BCG (6), measles-rubella (4) and inactivated polio vaccine (2) were lower than their vaccine vial size of 20, 10, and 5 doses, respectively. The wastage was high due to smaller session size and larger vial size, necessitating the disposal of unused doses. Better micro-planning to increase the session size and procuring vaccines with smaller vial sizes needs to be tested as a strategy to reduce vaccine wastage.

## 1. Introduction

Immunization is a powerful and cost-effective public health tool estimated to save about 2 to 3 million lives every year [1]. Immunization averts vaccine-preventable diseases (VPD), and benefits indirectly through improving cognitive development, educational attainment, labour productivity, income generation, savings and investment. A study estimated that in low-income countries, for every dollar spent on immunization, 16 dollars are expected to be saved in healthcare costs, lost wages, and lost productivity due to illness and death [2].

Immunization programs today reach over 85% of the world’s children [1]. It is estimated that immunizing all the children worldwide can further avert 1.5 million childhood deaths [1]. In this regard, 194 countries around the globe endorsed a shared vision known as “Decade of Vaccine” (DoV) to spread out the benefits of immunization to every person by 2020 [3]. The DoV collaboration developed a Global Vaccine Action Plan (GVAP) to improve the immunization programmes in partner countries [4]. The GVAP urged national immunization programmes to improve the supply chain mechanisms and ensure effective vaccine utilization by minimizing vaccine wastage [4].

Effective vaccine utilization is an integral component of vaccine security. Vaccine wastage can occur during procurement, transport, and storage due to breakage and spoilage due to cold chain failure, or expiry. Vaccine wastage at the service delivery point (immunization sites) is due to either pilferage during reconstitution and vaccination or low session size (number of beneficiaries availing vaccines during the session). High vaccine wastage inflates vaccine demand and increases unnecessary vaccine procurement and supply chain costs. Vaccine wastage is one of the key factors to be considered for vaccine forecasting, and needs estimations to avoid stock-outs or overstocks [5].

Globally, observed wastage rates range between 15% to 50% for lyophilized vaccines and 5% to 25% for liquid vaccines [6]. However, studies from various low-and middle-income countries have reported high rates of vaccine wastage [7,8,9,10]. As vaccine wastage rate depends on session size, vial size, and formulation, it varies across the countries and even within the countries [7,8,10]. The systematic assessment of vaccine wastage and session size during immunization can help in deciding on optimal vial size to be procured under the immunization programme, in order to limit vaccine wastage [10].

In Myanmar, the Expanded Programme on Immunization (EPI) covers about one million beneficiaries every year through routine immunization. The National Strategic Plan (NSP) for immunization intends to achieve 90% vaccine coverage by 2021. In order to achieve high coverage, it is projected that the programme has to spend about USD $126 million from 2017 to 2021 [11]. In order to reduce the expenditure, the NSP recommended enhancing vaccine utilization by minimizing the vaccine wastage without jeopardizing vaccine coverage [12].

Currently, in Myanmar, the maximum indicative vaccine wastage rates for each vaccine are used for vaccine forecasting at the national level. Though it is important to assess and monitor the vaccine wastage rate, there has been no systematic assessment of vaccine wastage. Also, there is a paucity of information on session size at immunization sites, which determines the optimal vial size for vaccine procurement.

Hence, in selected twenty subcenters (service delivery points) of the Mandalay region of Myanmar during 2018, we aimed to assess (1) vaccine wastage rate for vaccines delivered through EPI programme; (2) variations in vaccine wastage rate with characteristics of vaccine, session size, and month of activity; (3) the median session size for each of the vaccines.

## 2. Materials and Methods

### 2.1. Study Design

A cross-sectional descriptive study was conducted using secondary data routinely collected by the EPI of Myanmar.

### 2.2. Study Setting

#### 2.2.1. General Setting

Myanmar is situated between China and India, with a population of 51.5 million and administratively divided into 14 states and regions. The population density is approximately 79 people per square kilometer. The estimated birth rate is 18.1 births per 1000 population. The highest population density is seen in Yangon and Mandalay regions, with about one-fourth of the total population residing in these regions.

#### 2.2.2. Specific Setting

##### Expanded Programme on Immunization

The EPI was started in 1978 under the Ministry of Health and Sports (MOHS). Since its inception, Bacillus Calmette–Guérin (BCG), Diphtheria–Tetanus–Pertussis (DTP), and tetanus toxoid (TT) vaccines were provided free of cost through routine immunization session in public health facilities. Newer vaccines were added to EPI in 1978. Currently, the EPI programme delivers nine vaccines, namely BCG, pentavalent vaccine, oral polio vaccine (OPV), injectable polio vaccine (IPV), pneumococcal conjugate vaccine (PCV), measles–rubella (MR), Japanese encephalitis (JE), hepatitis B, and tetanus–diphtheria (Td). The details on the formulation, mode of administration, timing, and number of doses in each vial are described in Table 1.

##### Vaccine Procurement, Storage, and Distribution

The annual vaccine requirement under the EPI is estimated at the national level during September of every year, and procured through the United Nations Children’s Fund (UNICEF) supply division. Since 2017, the IPV supply is entirely supported by the Global Alliance for Vaccines and Immunizations (GAVI). The rest are procured either solely by MOHS (BCG, OPV, MR, Td, and hepatitis B) or in co-financing with GAVI (pentavalent, JE, and PCV). According to the quarterly shipment plan, from the UNICEF supply division, the vaccines are delivered to Central Cold Room (CCR) at Yangon. The vaccines are then distributed to sub-national vaccine stores, township vaccine stores, and rural health centers (RHCs) for storage with cold chain monitoring. These vaccine stores dispense the vaccines to service delivery points, like subcentres, based on the request from the midwife conducting the immunization session.

##### Service Delivery by Midwife at Subcentre

Midwives estimates the vaccine requirement for a month based on the duelist of eligible children and the number of sessions planned for a month. In the first week of every month, either fixed immunization sessions at the health facility or the outreach sessions are conducted in the community. Usually, one fixed immunization session is conducted at the subcentre. The outreach sessions are conducted in each of the villages (about four or five) under the subcentre. If there are no beneficiaries for the particular village in the month, then the session for that village is skipped. In total, five to six immunization sessions are conducted through each subcentre per month during the first week. The session plan is documented in the micro-plan maintained by the midwife.

The midwife collects the required number of vaccine vials for the whole month from the RHC on the day of the first session of the month, or a day before the same. The vaccines are carried in vaccine carriers with four ice packs. The vaccine vial is opened even if there is a single beneficiary to be immunized during the session. The multi-dose vial policy is followed, and the vaccines that can be used during multiple sessions are carried to the next session in the subcentre. The midwife discards those vaccines that cannot be carried to the next session. During the whole week, the cold chain is maintained in vaccine carriers using ice packs. In the last session of the subcentre, all the unused vaccines are discarded. The unopened vaccine vials, if any, are returned to RHC for storing in the cold chain.

##### Recording and Reporting

During each session, the midwife documents the number of vials/doses taken per session, as well as the number of doses administered to the beneficiaries for each vaccine in the paper-based “tally sheet”. Every month, the midwife, under the supervision of the health assistant (HA) from the RHC, prepares and submits the monthly “compilation form” listing details of all the sessions conducted in the month.

### 2.3. Study Site and Sampling

Based on convenience, the Mandalay region was considered for this study, as the region had better data documentation and reporting compared to other regions. We included 20 subcentres in the region. No sample size was calculated, and the number of subcenters was based on the feasibility of collecting data.

Two-stage sampling was used to select the subcenters. We initially selected 20 out of 28 townships in the Mandalay region using computer-generated random numbers. Within each selected township, we used simple random sampling using the lottery method to select one subcentre. Details of all the sessions conducted during 2018 in the selected subcentre were included.

### 2.4. Data Variables and Sources of Data

The immunization session details, such as the month of activity, number of vials of each vaccine opened, number of doses of each vaccine in an opened vial, and the number of doses of each vaccine administered, was extracted from the “compilation form” maintained by the midwife. The ANO (principal investigator) extracted the data in July 2019 using a structured data extraction proforma.

### 2.5. Data Analysis and Statistics

Data was entered and analyzed using Microsoft Excel 2016. The total number of vaccine doses in the opened vials and the total number of doses administered in the 20 subcenters in the year 2018 were deduced by adding reported values from each session. The vaccine wastage rate at the service delivery point for each vaccine in the Mandalay region for 2018 was calculated using the following formula: (total number of doses in open vial – total number of doses administered)/total number of doses in open vial) × 100.

The vaccine wastage factor for each vaccine was calculated using the following formula: 100/(100 − vaccine wastage rate).

The vaccine wastage rates for each vaccine at each of the subcenters were calculated, and the distribution of these rates was summarized using median and inter-quartile range (IQR). The vaccine wastage rates and vaccine wastage factors were calculated by categorizing vaccines based on their specifications. Using OpenEpi online calculator, the difference in vaccine dose wastage across the vaccine characteristics was calculated, and the risk of wastage was expressed in the form of prevalence ratios with 95% confidence interval (CI). The vaccine wastage rates were calculated for each vaccine across the month of activity, and were depicted as a bar diagram.

Of the total sessions conducted in the 20 subcentres, the number and proportion of sessions with at least one beneficiary for the given vaccine were calculated. The median and interquartile range (IQR) was used to summarize the distribution of the number of beneficiaries for each vaccine per session when at least one beneficiary for the given vaccine is present.

### 2.6. Ethics Approval

The study was approved by the Institutional Review Board, Department of Medical Research, Myanmar (ERC#2019-63), and the Union Ethics Advisory Group, Paris, France (06/19). Permission from the Expanded Programme for Immunization (EPI) of Myanmar was obtained for accessing the routinely collected data.

## 3. Results

In total, 990 immunization sessions were conducted in 20 subcentres during 2018. The median (IQR) number of sessions conducted per month in the subcenters was 5 (2–6).

### 3.1. Session Size for Vaccines

Of the 990 immunization sessions, 378 (38.2%) sessions had at least one beneficiary for the BCG vaccine. The median (IQR) session size for the BCG in sessions with at least one beneficiary was 6 (2–8) beneficiaries. Of the total, 887 (89.6%) sessions had at least one beneficiary for OPV, pentavalent, and PCV, with the median (IQR) session size of 5 (2–9) beneficiaries. In 665 (67.2%) sessions with at least one beneficiary for IPV, the median session size for IPV was 2 (1–4) beneficiaries. The session size for the MR vaccine was 4 (2–8) beneficiaries among 688 (69.5%) sessions with at least one beneficiary for the MR vaccine. The median (IQR) session size for JE, hepatitis B, and Td vaccines were 3 (1–5), 8 (1–12), and 4 (2–7), respectively (Table 2).

### 3.2. Session Size for Vaccines

Table 3 shows the vaccine wastage rate and corresponding vaccine wastage factor for individual vaccines. Among all the vaccines, BCG had the highest vaccine wastage rate at 54.9%, and the hepatitis B vaccine had the lowest vaccine wastage rate at 1.5%. The vaccine wastage rate for OPV was 30.2%, IPV was 28.3%, MR vaccine was 27.4%, JE vaccine was 24.6%, Td was 20.4%, pentavalent vaccine was 18.7%, and PCV was 7.4%. The vaccine wastage factors ranged between 2.22 for BCG to 1.02 for the Hepatitis B vaccine.

### 3.3. Vaccine Wastage Rate Across Subcentres, Vaccine Specifications, and Month of Activity

**Figure 1:**
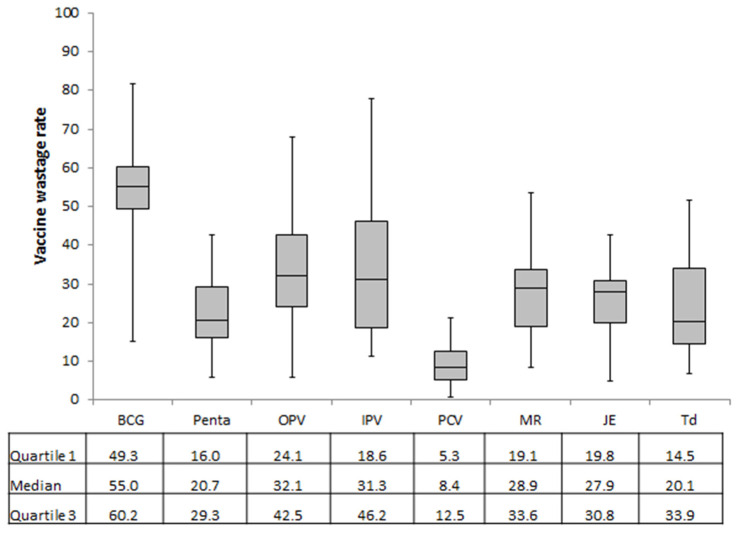
Distribution of vaccine wastage rates of vaccines delivered through the EPI across 20 subcentres (service delivery points) of the Mandalay region, Myanmar, in 2018. Abbreviations: EPI, Expanded Programme on Immunization; BCG, Bacillus Calmette–Guérin; OPV, oral polio vaccine; IPV, injectable polio vaccine; PCV, pneumococcal conjugate vaccine; MR, measles–rubella; JE, Japanese encephalitis; Td, tetanus–diphtheria; Hep B, hepatitis B.

The distribution of vaccine wastage rate across the 20 subcentres for each vaccine is depicted in Figure 1. The median (IQR) vaccine wastage rate for BCG, pentavalent, and IPV were 55.0% (49.3–60.2%), 20.7% (16.0–29.3%), and 31.3% (18.6–46.2%). During the study period, the hepatitis B vaccine was delivered only in two subcentres. Of the two, only one subcentre had vaccine wastage with a rate of 1.8%.

The lyophilized vaccines had a vaccine wastage rate of 36.9%, with 1.78 (95% CI: 1.73 to 1.84) times risk wastage compared to liquid vaccines. Those vaccines with 20 doses per vial had a vaccine wastage rate of 38.8% with 5.29 (95% CI: 4.86 to 5.77) times risk wastage compared to vaccines with fewer than five doses per vial. The vaccine wastage rate was 39.1% for vaccines that require a single dose per child, and 29.0% for those vaccines that require three doses complete immunization. The vaccine wastage rate of the multi-dose vial and non-multi-dose vial was 20.7% and 36.9%, respectively (Table 4).

**Figure 2:**
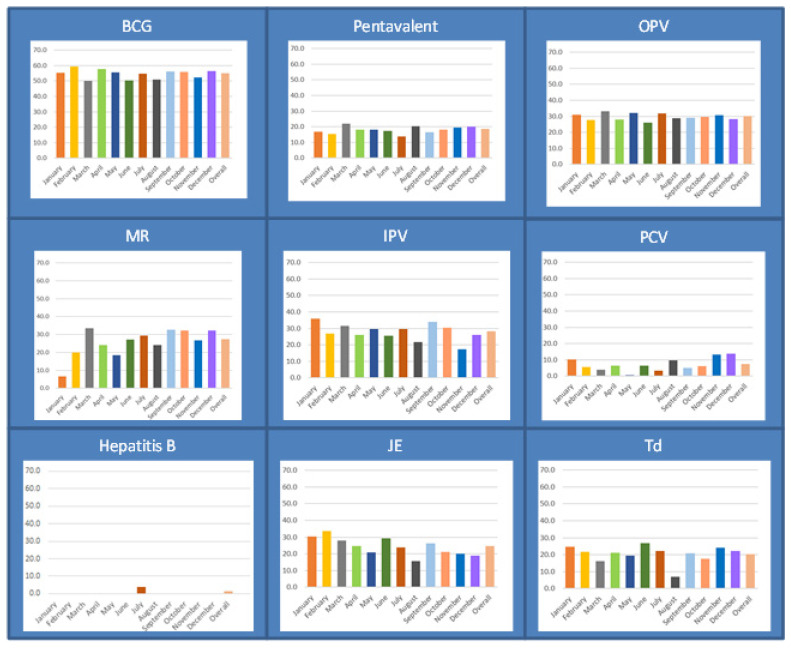
Vaccine wastage rates across of vaccines delivered through the EPI in 20 selected subcentres (service delivery points) of the Mandalay region, Myanmar, in 2018. Abbreviations: EPI, Expanded Programme on Immunization; BCG, Bacillus Calmette–Guérin; OPV, oral polio vaccine; IPV, injectable polio vaccine; PCV, pneumococcal conjugate vaccine; MR, measles–rubella; JE, Japanese encephalitis; Td, tetanus–diphtheria; Hep B, hepatitis B.

Figure 2 shows the vaccine wastage rate for individual vaccines across the month of activity. None of the vaccines showed any pattern in vaccine wastage rates across the month of activity.

## 4. Discussion

This is the first study to have assessed the immunization session size and vaccine wastage rate at service delivery points for vaccines delivered under the EPI of Myanmar. The study has three key findings. First, the immunization session sizes were small compared to the vial size of the vaccines used in the programme. Second, the vaccine wastage rates were higher than the indicative WHO rates for BCG, IPV, and MR vaccines. Also, the vaccine wastage occurred throughout the year, without any difference across the months. Third, the vaccine wastage was high in vaccines with larger vial size and those requiring only a single dose of immunization.

The study findings have to be interpreted in light of the limitations listed here. First, the study was conducted only in Mandalay region, with relatively better accessibility (plain area) to health facilities, higher population density, and vaccine coverage rates compared to other states and regions. Thus, the study result cannot be generalized to the whole of Myanmar, as the calculated vaccine wastage rates could be lower than that in other states and regions of the country. Second, we did not include immunization service delivery points of urban areas (urban health centres and tertiary hospitals), which might have had a larger session size and lower vaccine wastage rate compared to subcentres. Thus, we could have overestimated the vaccine wastage rates. However, we assume that the inclusion of only subcentres might not have largely affected the rates calculated for the region, as about 80% of the beneficiaries receive immunization through subcentres. Third, due to deficiencies in programme documentation, we could not assess “closed vial wastage” due to expiry, breakage, or spoilage. Thus, the wastage rates calculated here only reflect “open vial wastage”, due to either pilferage during administration or discarding of unused doses at the end of the session. Thus, the vaccine wastage rates calculated here could be an underestimate. Fourth, we aggregated the data over each month to calculate the vaccine wastage, given the limitation with the recording of data. Thus, we failed to calculate the vaccine wastage for each opened vaccine vial. However, a calculation based on one vaccine vial per session and the number of beneficiaries per session would have grossly overestimated the wastage rates, as the opened vaccine vials were carried across the sessions. However, we cannot estimate the net effect of these limitations on the calculated vaccine wastage rate.

In the current study, the vaccine wastage rate for the BCG vaccine was higher than the WHO’s indicated level of 50%, yet was within the vaccine wastage rate (77%) used for vaccine forecasting in Myanmar. A vaccine wastage assessment by UNICEF and a few studies other studies from India have reported relatively higher wastage for BCG (60–77%) [10,13,14]. The difference in the immunization schedule and service delivery points between the countries might be the reason for such variation. In India, BCG is administered immediately after birth. Thus, the session size for BCG at subcentres is smaller, as the majority of the beneficiaries are immunized at the institutions where they were delivered. However, the EPI of Myanmar recommends the administration of BCG within one year after birth, and this helps to accumulate more children belonging to a wider range of birth cohorts, and thus more beneficiaries. This adjustment is evident, as a BCG vial was opened only in about 4 out of 10 immunization sessions in the current study. On the contrary, the IPV, which is administered as a single dose in the fourth month, had a high wastage rate despite having a vial size of only five. This is because, to ensure the timeliness of the vaccination, IPV was administered at the fourth month, requiring more sessions than BCG (68% vs 38%) and leading to smaller session sizes.

Though the vial size of OPV was the same as BCG (20 doses), the wastage rate was less. The potential reason for such a lower wastage rate is due to the administration of three doses of OPV for complete immunization. This inflates the total number of beneficiaries by three times compared to BCG, and thus reduces the wastage rate, due to larger session size.

The current study reported high wastage for oral, lyophilized, and multi-dose vaccines. Similar results have also been observed in studies from various parts of the world [10,15,16,17,18]. The current findings support that multi-dose vials with a multi-dose vial policy has less wastage compared to those without a multi-dose vial policy. In the current study, vials with more than 20 doses had more wastage compared to lower dose vials. Also, the wastage was less in vaccines with multiple dosage requirements, such as PCV, pentavalent, MR, and Td, compared to vaccines that are administered only once. The number of beneficiaries at each session site plays a major role in determine wastage in this situation.

The study has a few strengths. First, we conducted the study using routinely programmatic data, reflecting on-the-ground reality. Second, we used a simple random sampling method for the selection of subcentres within the Mandalay region, thus reducing potential selection bias. Third, we included all the sessions conducted over one year. Thus, the calculated vaccine wastage rate was immune to seasonal variations in temperature, number of immunization sessions per month, and session sizes.

Our study findings have some implications for the EPI. First, the vial size of the vaccines delivered under the EPI is larger than the average immunization session size, leading to high vaccine wastage. Currently, the programme is procuring 20 dose vials of OPV, five dose vials of IPV, and 10 dose vials of MR vaccines. In the global market, lower vial sizes of OPV (10 doses), IPV (1 dose per vial), and MR vaccines (5 doses) are available. The programme can procure the vaccines with smaller vial sizes when available to limit vaccine wastage. However, recent modeling studies have shown that while a change to smaller vials could reduce wastage, this might not reduce overall costs, due to higher manufacturing and storage costs. Alternatively, the EPI can procure each vaccine with varied vial sizes and deliver the vaccines with small vial sizes to immunization sessions through subcentres with small session sizes. However, prior to such procurement of vaccines of smaller vial sizes, a thorough assessment of cold chain capacity and cost implications needs to be conducted. As an initial step, the simulations with information from this study (session size and wastage rates), cold chain capacity, and monetary resources available in the country can be conducted to deduce a procurement plan.

Second, the immunization session sizes are small and lead to high vaccine wastage rates compared to the WHO’s indicative rates. There is a need for deducing efficient micro-plans at the subcentre level, in order to increase the number of beneficiaries per opened vaccine vial without compromising vaccine coverage and timeliness. Conducting only fixed static immunization sessions at subcentres and requesting all the beneficiaries of catchment villages to visit such sessions could reduce the vaccine coverage. Similarly, trying to accumulate the beneficiaries over two to three months to ensure an adequate number of beneficiaries might lead to delayed immunization and create a window period where a child could be prone to VPD. Currently, the monthly outreach immunization sessions at catchment villages are scheduled over three to four days, requiring the opening of new vaccine vials. Alternatively, these sessions can be conducted within a single day at different time slots, and then a single open vaccine vial can be used in multiple villages. However, transportation arrangements need to be made available for the midwife to reach the villages by the scheduled time for carrying out sessions.

Third, to monitor vaccine wastage at the national, sub-national, and regional levels, there is a need to establish a robust recording system to deduce vaccine wastage rates. With the existing recording and reporting formats of the EPI, we could calculate vaccine wastage rates only due to “open vial wastage”, but not “closed vial wastage”. There is no recording system to document the details of the number of vaccine vials expired, broken, spoiled, and lost at various levels of storage and transportation. In line with the study findings, the EPI has initiated piloting a logistics and management information system (LMIS), which can capture vaccine pilferage details at storage facilities and service delivery points. The EPI programme managers have also been trained to deduce vaccine wastage rates at state and regional levels using routine data. In the future, the EPI has to constitute a research study, using LMIS data to comprehensively determine vaccine wastage (both open vial and closed vial wastage), and also to assess the factors associated with such wastage to enhance vaccine utilization.

## 5. Conclusions

The vaccine wastage rates were higher than the WHO’s indicative rates for BCG, IPV, and MR vaccines at the service delivery point. The wastage rates were high due to relatively large vial sizes compared to session size. Better micro-planning by scheduling multiple sessions on a single day to increase session size and procuring vaccines with smaller vial sizes can be tested to reduce vaccine wastage and costs.

## Figures and Tables

**Table 1:** Specification of vaccines delivered under the Expanded Programme on Immunization (EPI) in Myanmar during 2018.

Vaccine	Vaccine Type	Number of Doses per Vial	Mode of Administration	Timing Since Birth	Number of Doses per Beneficiary	Indicative Vaccine Wastage Rate
BCG	Lyophilized	20	Injectable	Within 1 year	1	50%
Pentavalent	Liquid	10	Injectable	Second, fourth, and sixth month	3	25%
OPV	Liquid	20	Oral	Second, fourth, and sixth month	3	40%
IPV	Liquid	5	Injectable	Fourth month	1	10%
PCV	Liquid	4	Injectable	Second, fourth, and sixth month	3	10%
MR	Lyophilized	10	Injectable	Ninth and 18th month	2	25%
JE	Lyophilized	5	Injectable	Ninth month	1	10%
Hepatitis-B	Liquid	1	Injectable	At birth	1	5%
Td	Liquid	10	Injectable	Pregnant women at one-month intervals	2	25%

Abbreviations: BCG, Bacillus Calmette–Guérin; OPV, oral polio vaccine; IPV, injectable polio vaccine; PCV, pneumococcal conjugate vaccine; MR, measles–rubella; JE, Japanese encephalitis; Td, tetanus diphtheria; Hep B, Hepatitis B.

**Table 2:** Number and proportion of sessions with at least one beneficiary and median number of beneficiaries per session for each vaccine during immunization sessions, conducted in 20 subcentres of the Mandalay region, Myanmar, in 2018 (*n* = 990).

Vaccine	Sessions with at Least One Beneficiary		Median (IQR) *
	*n*	(%) *	
BCG	378	(38.2)	6 (2–8)
Pentavalent	887	(89.6)	5 (2–9)
OPV	887	(89.6)	5 (2–9)
IPV	665	(67.2)	2 (1–4)
PCV	887	(89.6)	5 (2–9)
MR	688	(69.5)	4 (2–8)
JE	566	(57.2)	3 (1–5)
Hep B	12	(1.2)	9 (8–12)
Td	794	(80.2)	4 (2–7)

* Proportion and median calculated with 990 sessions as the denominator. Abbreviations: EPI, Expanded Programme on Immunization; BCG, Bacillus Calmette–Guérin; OPV, oral polio vaccine; IPV, injectable polio vaccine; PCV, pneumococcal conjugate vaccine; MR, measles–rubella; JE, Japanese encephalitis; Td, tetanus–diphtheria; Hep B, hepatitis B.

**Table 3:** Vaccine wastage rates and vaccine wastage factor of vaccines delivered under EPI in 20 subcentres (service delivery points) of the Mandalay region, Myanmar, in 2018.

Vaccine	Number Doses in Opened Vial (A)	Number of Doses Administered (B)	Number of Doses Wasted (C) *	Vaccine Wastage Rate (D) ^#^	Vaccine Wastage Factor (E) ^$^
BCG	4840	2182	2658	54.9	2.22
Penta	7880	6404	1476	18.7	1.23
OPV	9140	6378	2762	30.2	1.43
IPV	3035	2176	859	28.3	1.39
PCV	6832	6324	508	7.4	1.08
MR	5770	4191	1579	27.4	1.38
JE	2680	2020	660	24.6	1.33
HepB	134	132	2	1.5	1.02
Td	5320	4234	1086	20.4	1.26

* C = A − B; ^#^ D = (C/A) × 100; ^$^ E = 100/(100 − D). Abbreviations: EPI, Expanded Programme on Immunization; BCG, Bacillus Calmette–Guérin; OPV, oral polio vaccine; IPV, injectable polio vaccine; PCV, pneumococcal conjugate vaccine; MR, measles–rubella; JE, Japanese encephalitis; Td, tetanus–diphtheria; Hep B, hepatitis B.

**Table 4:** Vaccine wastage rates and vaccine wastage factor of vaccines across various specifications of vaccines delivered under the EPI in 20 subcentres (service delivery points) of the Mandalay region, Myanmar, in 2018.

Vaccine	Number Doses in Opened Vial (A)	Number of Doses Administered (B)	Number of Doses Wasted (C) *	Vaccine Wastage Rate (D) ^#^	PR (95% CI) ^$^
**Mode of Administration**					
Oral vaccine (OPV)	9140	6378	2762	30.2	1.25 (1.20–1.29)
Injectable vaccine (BCG, IPV, MR, Td, JE, Pentavalent, PCV, Hep B)	36,491	27,663	8828	24.2	1
**Composition of Vaccine**					
Lyophilized (BCG, MR and JE)	13,290	8393	4897	36.9	1.78 (1.73–1.84)
Liquid (OPV, PCV, Td, IPV, Pentavalent, Hep B)	32,341	25,648	6693	20.7	1
**Multi-Dose Vial Policy**					
Yes (OPV, Pentavalent, Td, IPV, PCV, Hep B)	32,341	25,648	6693	20.7	1
No (BCG, JE, MR)	13,290	8393	4897	36.9	1.78 (1.73–1.84)
**Number of Doses in a Vial**					
<5 (PCV, Hep B)	6966	6456	510	7.3	1
5 (IPV, JE)	5715	4196	1519	26.5	3.63 (3.30–3.99)
10 (Pentavalent, MR, Td)	18,970	14,829	4141	21.8	2.98 (2.73–3.25)
20 (OPV, BCG)	13,980	8560	5420	38.8	5.29 (4.86–5.77)
**Number of Doses per Child**					
1 (BCG, IPV, JE, Hep B))	10,689	6510	4179	39.1	1.35 (1.30–1.39)
2 (MR)	5770	4191	1579	27.4	0.94 (0.90–0.99)
3 (Pentavalent, OPV, PCV)	17,945	12,745	5200	29.0	1

* C = A − B; ^#^ D = (C/A) × 100; ^$^ PR calculated with number of doses wasted as an outcome of interest. Abbreviations: PR, prevalence ratio; CI, Confidence Interval; BCG, Bacillus Calmette–Guérin; OPV, oral polio vaccine; IPV, injectable polio vaccine; PCV, pneumococcal conjugate vaccine; MR, measles–rubella; JE, Japanese encephalitis; Td, tetanus diphtheria; Hep B, hepatitis B.
